# Role of sex in the association between childhood socioeconomic position and cognitive ageing in later life

**DOI:** 10.1038/s41598-021-84022-1

**Published:** 2021-02-25

**Authors:** Katrin Wolfova, Zsofia Csajbok, Anna Kagstrom, Ingemar Kåreholt, Pavla Cermakova

**Affiliations:** 1 Department of Psychiatry and Medical Psychology, Third Faculty of Medicine, Charles University in Prague, Ruska 87, 100 00 Prague 10, Czech Republic; 2 National Institute of Mental Health, Topolova 748, 250 67 Klecany, Czech Republic; 3 Department of Philosophy and History of Science, Faculty of Science, Charles University in Prague, Vinicna 7, 128 00 Prague, Czech Republic; 4 Institute of Gerontology, Aging Research Network - Jönköping (ARN-J), School of Health and Welfare, Jönköping University, Jönköping, Sweden; 5 Aging Research Center, Karolinska Institutet and Stockholm University, Stockholm, Sweden; 6 Department of Epidemiology, Second Faculty of Medicine, Charles University in Prague, Plzenska 130/221, 150 00 Prague, Czech Republic

**Keywords:** Neuroscience, Neurology, Risk factors

## Abstract

We aimed to explore sex differences in the association of childhood socioeconomic position (SEP) with the level of cognitive performance and the rate of cognitive decline. We studied 84,059 individuals (55% women; mean age 64 years) from the Survey on Health, Ageing and Retirement in Europe. Sex differences in the association of childhood SEP (household characteristics at age 10) with the level of cognitive performance (verbal fluency, immediate recall, delayed recall) were analysed using multilevel linear regression. Structural equation modelling tested education, depressive symptoms and physical state as mediators. The relationship between childhood socioeconomic advantage and disadvantage and the rate of cognitive decline was assessed using linear mixed-effects models. Higher childhood SEP was associated with a higher level of cognitive performance to a greater extent in women (B = 0.122; 95% CI 0.092–0.151) than in men (B = 0.109; 95% CI 0.084–0.135). The strongest mediator was education. Childhood socioeconomic disadvantage was related to a higher rate of decline in delayed recall in both sexes, with a greater association in women. Strategies to prevent impaired late-life cognitive functioning, such as reducing childhood socioeconomic disadvantages and improving education, might have a greater benefit for women.

## Introduction

Cognitive impairment, which primarily occurs with advanced age^1^, affects women to a larger extent than men^2^. Given the multifactorial aetiology of this syndrome, longer life expectancy of women can only partially explain this gap. Increasing evidence suggests that the roots of cognitive impairment may lie in childhood socioeconomic conditions. In previous studies, growing up in a crowded household and less cognitively stimulating environment have been consistently associated with lower levels of cognitive performance in late life and increased risk of dementia^3,4^. However, there is less consensus on whether childhood socioeconomic position (SEP) additionally influences the rate of cognitive decline^5^.

A growing body of literature indicates that experiencing socioeconomic adversity in childhood has stronger consequences on the health of women than men. For example, the effects of childhood poverty on obesity and cardiovascular diseases are disproportionately stronger for women^6,7^. It is unclear whether these sex differences exist also for cognitive health. While mechanisms underlying sex differences related to socioeconomic adversity are likely a complex combination of biopsychosocial variables experienced across the life course, the direction and nature of the relationships of SEP, sex and health have yet to be fully understood.

Scientists have long sought understanding of the nature of discrepancies found in sex and cognition^8^. While early research observed sex differences across a range of cognitive measures, modern science challenges the appropriateness of conclusions of globally intrinsic sex differences in cognition^9,10^. Theoretical challenges against concrete sex differences in cognition align with principles from sociocultural theory; which posits that a society’s cultural structure around sex related restrictions and opportunities drive cognitive differences of men and women^11^. This theory implies that when socio-cultural variables are equal across sexes, the cognitive abilities of men and women have more parity than previously thought^8^.

SEP affects our overall physical and mental functioning, and is associated with health-related risk and protective factors including educational achievement. Cultural expectations of men’s and women’s roles interact with behaviour through social, psychological, and biological processes through encouragement of stereotypical sex-related behaviours as well as hormonal, reward, and cardiovascular mechanisms, which all contribute to differences in men’s and women’s behaviour development across the life course^11^. Childhood SEP can impact a range of risk factors over the life-course including, psychological and physical health, and health-predicting behaviours and influences, and psychological health differences between sexes are predictable in large by inequalities in respective societies^12^. As a result, women have fewer resources available throughout their lives that would provide them with opportunities for building and maintaining resilience^13^.

In addition, previous findings show that exposure to early-life stressors such as socioeconomic hardship leads to a dysregulation of stress response^14^. Considering that exposure to elevated stress hormone levels causes thinning of several cortical areas to a greater extent in females than males^15–17^, it is plausible to hypothesize that experiencing childhood socioeconomic disadvantage could have a larger effect on the cognition of women than men. Reducing the effects of childhood socioeconomic disadvantage through targeted preventative strategies could therefore contribute to narrowing sex disparities related to the burden of lower cognitive functioning. In a diverse population of older adults residing in 21 different countries, the present study aimed to explore sex differences in (1) the association of childhood SEP and the level of cognitive performance, (2) mediators in this association and (3) the relationship between childhood SEP and the rate of cognitive decline.

## Results

### Cross-sectional analysis

Women scored higher in cognitive tests (0.03 ± 0.85) than men (− 0.04 ± 0.80; < 0.001, Cohen’s d =  − 0.092), but there was no difference in their childhood SEP (Table 1). Higher childhood SEP was associated with a higher level of cognitive performance in all models in the whole sample (Supplementary Table S1). There was an interaction between childhood SEP and sex (*p* < 0.001) in all models (Table 2). When stratified by sex and adjusted for age, higher childhood SEP was significantly associated with a higher level of cognitive performance in both sexes, but to a larger extent in women (B = 0.238; 95% confidence interval (CI) 0.203–0.271) than in men (B = 0.208; 95% CI 0.180–0.235; Table 2, Model 1).

**Table 1:** Sex differences in baseline characteristics.

	Women (n = 46,148)	Men (n = 37,911)	Sex difference (*p* value)	Effect size
Cognition (z-score), mean ± SD	0.03 ± 0.85	− 0.04 ± 0.80	< 0.001	− 0.092
Verbal fluency, mean ± SD	19.92 ± 7.67	20.18 ± 7.45	< 0.001	0.034
Immediate recall, mean ± SD	5.30 ± 1.81	5.09 ± 1.73	< 0.001	− 0.116
Delayed recall, mean ± SD	3.89 ± 2.15	3.59 ± 2.01	< 0.001	− 0.147
Childhood socioeconomic position, mean ± SD	0.00 ± 0.80	0.00 ± 0.80	0.649	0.003
Age, median (IQR)	62.00 (55.00–71.00)	62.00 (56.00–70.00)	0.694	0.003
Years of education, mean ± SD	10.47 ± 4.26	11.20 ± 4.40	< 0.001	0.169
Depressive symptoms, n (%)	10,043 (21.84)	4048 (10.72)	< 0.001	0.148
Highest decile of household net worth, n (%)^a^	4223 (9.15)	4181 (11.03)	< 0.001	− 0.031
Current working status, n (%)	13,312 (29.01)	13,968 (36.95)	< 0.001	− 0.084
Children: 2 and more, n (%)	33,622 (72.86)	27,848 (73.46)	0.051	− 0.007
Grandchildren: 2 and more, n (%)	25,145 (54.49)	18,122 (47.80)	< 0.001	0.067
Living with a partner, n (%)	30,467 (66.02)	30,730 (81.06)	< 0.001	− 0.168
Limitations in IADL: 1 and more, n (%)	9263 (20.07)	4569 (12.05)	< 0.001	0.108
Chronic diseases: 1 and more, n (%)	22,273 (48.27)	16,526 (43.60)	< 0.001	0.047
CVD, n (%)	26,340 (57.10)	22,347 (58.96)	< 0.001	− 0.019
Body mass index, mean ± SD	26.65 ± 4.99	27.17 ± 4.03	< 0.001	0.113
Physical inactivity, n (%)	4682 (10.15)	2904 (7.66)	< 0.001	0.043
Smoking, n (%)	16,040 (34.87)	22,860 (60.48)	< 0.001	− 0.256
Alcohol use, n (%)	4180 (9.06)	9146 (24.13)	< 0.001	− 0.205
Maximal grip strength, mean (± SD)	26.77 (7.16)	43.91 (10.25)	< 0.001	1.971

^a^Binary variable instead of continuous was used for household ratio for comprehensive interpretation of the results.

*SD* standard deviation, *IQR* interquartile range, *IADL* instrumental activities of daily living, *CVD* cardiovascular disease. Depressive symptoms are defined by 4 and more points on EURO-D scale. Alcohol use is defined as drinking more than 2 glasses of alcohol almost every day. Effect sizes are presented in Cramer’s V when Chi-square tests are employed on binary variables and in Cohen’s d when t-test have been used to compare continuous variables across sex.

**Table 2:** Association of childhood socioeconomic position with the level of cognitive performance.

	B (95% CI)		
	Women (n = 46,148)	Men (n = 37,911)	Interaction childhood SEP × sex in the whole sample
**Model 1**			
Childhood SEP	0.238 (0.203; 0.271)***	0.208 (0.180; 0.235)***	0.063 (0.052; 0.076)***
Between-country variance	0.070	0.042	
Within-country variance	0.476	0.474	
**Model 2**			
Childhood SEP	0.142 (0.110; 0.175)***	0.125 (0.097; 0.152)***	0.060 (0.048; 0.071)***
Between-country variance	0.056	0.034	
Within-country variance	0.443	0.445	
**Model 3**			
Childhood SEP	0.128 (0.098; 0.159)***	0.114 (0.087; 0.141)***	0.052 (0.040; 0.063)***
Between-country variance	0.051	0.032	
Within-country variance	0.429	0.428	
**Model 4**			
Childhood SEP	0.122 (0.092; 0.151)***	0.109 (0.084; 0.135)***	0.052 (0.041; 0.063)***
Between-country variance	0.043	0.026	
Within-country variance	0.411	0.409	

*p < 0.05, **p < 0.01, ***p < 0.001.

Results are unstandardized beta coefficients with 95% confidence intervals from multilevel regression models estimating the association of childhood socioeconomic position with cognition. *SEP* socioeconomic position, *CI* confidence interval.

*Model 1* adjusted for age.

*Model 2* adjusted for age and education.

*Model 3* adjusted for age, education, depressive symptoms and socioeconomic characteristics (household net worth, cohabitation status, number of children, number of grandchildren and current working status).

*Model 4* adjusted for age, education, socioeconomic characteristics (household net worth, cohabitation status, number of children, number of grandchildren, current working status) and health related characteristics (body mass index, depressive symptoms, limitations in instrumental activities of daily living, maximum of grip strength, physical inactivity, number of chronic diseases, smoking status, cardiovascular disease and alcohol use).

When adjusted also for education, the model attenuated by 40.3% in women and 39.9% in men but the association of childhood SEP with cognitive performance remained statistically significant in both sexes (women: B = 0.142; 95% CI 0.110–0.175 vs. men: B = 0.125; 95% CI 0.097–0.152; Table 2, Model 2). Adjusting for depressive symptoms and all sociodemographic characteristics additionally reduced the coefficients by 9.9% in women and 8.8% in men (Table 2, Model 3). When all sociodemographic and health-related covariates were entered, the coefficients attenuated further by approximately 4.6% in women and 4.4% in men (Table 2, Model 4). The differences between the coefficients in men and women were statistically significant in all models. The strongest predictor of cognitive performance in both sexes was education and the second strongest was age. Childhood SEP was the third strongest predictor in women and the fourth in men (Supplementary Table S2).

### Mediation analysis

**Figure 1:**
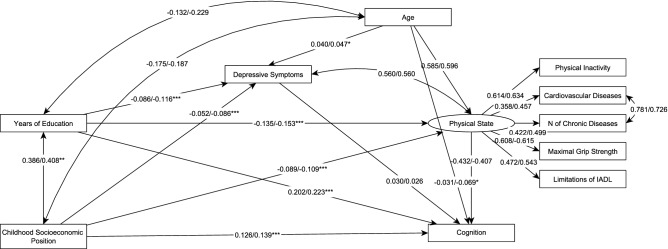
Multiple mediation model. *p < 0.05, **p < 0.01, ***p < 0.001. The figure presents the analysis testing the mediating effect of education, depressive symptoms, and the “physical state” latent factor on the association between childhood socioeconomic position and the level of cognitive performance controlled for age across sex. Standardized coefficients are presented on their respective arrows. Men’s and women’s coefficients are on the left and right position of the slash, respectively. All the paths are significant at *p* < 0.001. The paths significantly different between men and women are marked with respective asterisks. The whole mediation model explained 34.6% and 40.1% of men’s and women’s variance in cognition, respectively. The model accounted for data variance’s 14.9% and 16.6% in years of education in men and women, respectively. Depressive symptoms were explained up to 1.7% and 3.6% in men and women, respectively.

The mediation model tested the mediating effect of education, depressive symptoms and a “physical state” latent factor in the association of childhood SEP with the level of cognitive performance. The model had an acceptable fit against the data in both sexes (men: χ^2^(24) = 4457.627, root mean square error of approximation = 0.070 [90% CI 0.068–0.072], comparative fit index = 0.956, Tucker–Lewis index = 0.918; women: χ^2^(24) = 3327.370, root mean square error of approximation = 0.055 [90% CI 0.053–0.056], comparative fit index = 0.979, Tucker–Lewis index = 0.961). The model accounted for 40.1% of women’s and 34.6% of men’s variance in the level of cognitive performance (Fig. 1). Education was responsible for mediating 29.5% in men and 30.7% in women of the model’s total effect between childhood SEP and the level of cognitive performance. The effect was significantly stronger in women with a small effect size (d = 0.051, *p* < 0.001). Of the model’s total effect, “physical state” mediated 14.56% in men and 14.87% in women. The association was significantly stronger in women, however, with a very small effect size (d = 0.018, *p* < 0.01).

The mediating effects of depressive symptoms between childhood SEP and the level of cognitive performance were negative in both sexes and equally small, with virtually no difference (− 0.002 in both men and women). Education via the “physical state” was responsible for 8.43% of the model’s total effect in men and significantly stronger with 8.54% in women (d = 0.026, *p* < 0.001). Education via depressive symptoms had negative, though small, effects on cognition virtually equal across sex (-0.001 in both sexes). Childhood SEP had proportionally a stronger direct effect on the level of cognitive performance in men (48.28%) than in women (46.84%), however, the absolute effect was larger in women (0.148) than in men (0.126, d = 0.044, *p* < 0.001, see Supplementary Table S3 and Fig. S1 for unstandardized coefficients).

In sensitivity analysis, when physical inactivity and alcohol use were used instead of the “physical state” latent factor, the results showed that physical inactivity was a considerably weaker mediator than the “physical state” latent factor. Alcohol use mediated only a negligible amount of the overall effect of childhood SEP on the level of cognitive performance (Supplementary Figs. S2 and S3).

### Longitudinal analysis

Using the linear mixed-effect model adjusted for baseline age, we found a significant interaction between sex and childhood SEP in the association to the rate of decline in all three cognitive measures (*p* from likelihood ratio test < 0.001). Stratified by sex and using the binary variables childhood socioeconomic advantage and disadvantage, childhood socioeconomic disadvantage was related to a higher rate of decline in delayed recall in both sexes, but to a greater extent in women (women: B =  − 0.023; 95% CI  − 0.035; -0.011 vs. men: B =  − 0.018; 95% CI  − 0.032;  − 0.005, Table 3). Childhood socioeconomic disadvantage was not associated with a decline in immediate recall nor in verbal fluency in either sex. Childhood socioeconomic advantage was not related to the rate of decline in any of the measures in either sex (Table 4).

**Table 3:** Association of childhood socioeconomic disadvantage with the rate of cognitive decline.

	Women (n = 41,140)	Men (n = 33,139)
	B (95% CI)	B (95% CI)
**Delayed recall**		
Model 1	− 0.023 (− 0.035; − 0.011)**	− 0.018 (− 0.032; − 0.005)***
Model 2	− 0.022 (− 0.034; − 0.010)***	− 0.018 (− 0.032; − 0.005)**
Model 3	− 0.022 (− 0.034; − 0.010)***	− 0.018 (− 0.032; − 0.005)**
Model 4	− 0.020 (− 0.023; − 0.008)**	− 0.019 (− 0.032; − 0.005)**
**Immediate recall**		
Model 1	− 0.005 (− 0.015; 0.005)	0.003 (− 0.008; 0.015)
Model 2	− 0.004 (− 0.014; 0.006)	0.004 (− 0.008; 0.015)
Model 3	− 0.005 (− 0.015; 0.005)	0.003 (− 0.008; 0.015)
Model 4	− 0.004 (− 0.014; 0.006)	0.003 (− 0.009; 0.014)
**Verbal fluency**		
Model 1	− 0.034 (− 0.074; 0.007)	− 0.030 (− 0.079; 0.019)
Model 2	− 0.032 (− 0.073; 0.008)	− 0.030 (− 0.079; 0.018)
Model 3	− 0.031 (− 0.072; 0.010)	− 0.034 (− 0.082; 0.015)
Model 4	− 0.030 (− 0.071; 0.011)	− 0.033 (− 0.082; 0.016)

*p < 0.05, **p < 0.01, ***p < 0.001.

*Model 1* adjusted for age and practice effect.

*Model 2* adjusted for age, practice effect and education.

*Model 3* adjusted for age, practice effect, education, depressive symptoms and socioeconomic characteristics (household net worth, cohabitation status, number of children, number of grandchildren and current working status).

*Model 4* adjusted for age, practice effect, education, socioeconomic characteristics (household net worth, cohabitation status, number of children, number of grandchildren, current working status) and health related characteristics (body mass index, depressive symptoms, limitations in instrumental activities of daily living, maximum of grip strength, physical inactivity, number of chronic diseases, smoking status, cardiovascular disease and alcohol use).

**Table 4:** Association of childhood socioeconomic advantage with the rate of cognitive decline.

	Women (n = 41,140)	Men (n = 33,139)
	B (95% CI)	B (95% CI)
**Delayed recall**		
Model 1	0.024 (− 0.006; 0.053)	0.021 (− 0.013; 0.053)
Model 2	0.022 (− 0.010; 0.054)	0.018 (− 0.015; 0.051)
Model 3	0.021 (− 0.009; 0.051)	0.019 (− 0.013; 0.052)
Model 4	0.021 (− 0.009; 0.050)	0.020 (− 0.012; 0.053)
**Immediate recall**		
Model 1	0.022 (− 0.003; 0.047)	0.003 (− 0.026; 0.031)
Model 2	0.019 (− 0.006; 0.044)	0.001 (− 0.027; 0.029)
Model 3	0.019 (− 0.006; 0.044)	0.002 (− 0.026; 0.030)
Model 4	0.017 (− 0.007; 0.042)	0.004 (− 0.024; 0.032)
**Verbal fluency**		
Model 1	0.060 (− 0.045; 0.165)	− 0.006 (− 0.128; 0.117)
Model 2	0.051 (− 0.054; 0.156)	− 0.019 (− 0.141; 0.103)
Model 3	0.053 (− 0.051; 0.158)	− 0.014 (− 0.136; 0.108)
Model 4	0.052 (− 0.053; 0.156)	− 0.012 (− 0.134; 0.110)

*p < 0.05, **p < 0.01, ***p < 0.001.

*Model 1* adjusted for age and practice effect.

*Model 2* adjusted for age, practice effect and education.

*Model 3* adjusted for age, practice effect, education, depressive symptoms and socioeconomic characteristics (household net worth, cohabitation status, number of children, number of grandchildren and current working status).

*Model 4* adjusted for age, practice effect, education, socioeconomic characteristics (household net worth, cohabitation status, number of children, number of grandchildren, current working status) and health related characteristics (body mass index, depressive symptoms, limitations in instrumental activities of daily living, maximum of grip strength, physical inactivity, number of chronic diseases, smoking status, cardiovascular disease and alcohol use).

### Sensitivity analysis

Using an alternative measure of childhood SEP, we found a significant difference in the association of being in disadvantaged and the most disadvantaged childhood SEP with the level of cognitive performance between men and women in all four models. Stratifying by sex and using the most advantaged group as a reference category, there was a strong socioeconomic gradient across all four entries (disadvantaged, middle, advantaged, and most advantaged) in the level of cognitive performance in Model 1 in both women and men (Supplementary Table S4). These associations largely attenuated after adjustment for education; and being men in the advantaged group was no longer associated with lower level of cognitive performance in comparison to the most advantaged. After controlling for other socioeconomic and health- related characteristics, these associations attenuated, but did not lose their statistical significance.

## Discussion

This multi-centre population-based cohort study, capitalizing on a sample of more than 80,000 well-characterized individuals residing in 20 European countries and Israel, suggests that experiencing socioeconomic hardship in childhood could have a greater effect on cognitive health of women than of men. The sex difference in the relationship between childhood SEP and the level of cognitive performance was not explained by other socioeconomic and health related risk factors. Education was the strongest mediator of the association of childhood SEP with the level of cognitive performance. However, sex differences in the effects of childhood SEP on the rate of cognitive decline are less clear as we found only an association between childhood socioeconomic disadvantage and the rate of cognitive decline in delayed recall. These findings are of interest in the context of identifying modifiable early-life predictors of later-life cognitive performance, bringing improved understandings of discrepancies related to SEP, education and cognitive health across sexes. We suggest that preventing childhood socioeconomic adversity and improving educational opportunities could help to decrease the disproportionate burden of later-life lowered cognitive functioning in women.

This study is in line with literature suggesting that childhood socioeconomic adversity has stronger consequences on the health of women compared to men^18,19^. There is a substantial body of evidence surrounding sex discrepancies in the origin of cardiovascular disease, with several studies reporting stronger associations of childhood SEP with obesity, hypertension and risk of myocardial infarction in women than in men^6,20–22^. The implications of poor childhood SEP on later-life cognitive functioning and differences across sexes have previously been examined in two studies based on samples from the United Kingdom and the Unites States^19,23,24^. In a British birth cohort, Hurst and colleagues examined the influence of childhood SEP on nine markers of physical and cognitive performance^23^. They found a statistically significant sex difference in the association of childhood SEP with reaction time only, reporting a small interaction in magnitude, which indicated a slightly stronger association for men than for women^23^. In an American cohort, Lyu found that the association of childhood SEP with the level of memory was stronger for men than for women^24^.

Our findings challenge results from these previous studies^23,24^, suggesting that the consequences of childhood SEP related to cognitive performance are stronger for women than for men. Higher survival amongst women has been used as a dominant reason for a higher burden of cognitive disorders in women^25^. We propose that the heightened consequences of childhood socioeconomic adversity on women’s cognitive health also contributes to sex disparities. The modifying effect of sex on the relation between childhood socioeconomic adversity and poor cognitive outcome in later life might be explained by underlying biological pathways of sex-specific response to early life stressors. Low childhood SEP is an adverse experience that leads to hypothalamic–pituitary–adrenal axis dysregulation^14^. Exposure to the elevated corticotropin releasing hormone during early-life periods is a predictor of thinning of selective cortical regions related to cognition, and this association seems to be stronger in females^15–17^. As a consequence, women exposed to socioeconomic hardship might have lower cognitive reserve than men, increasing their risk of diminished cognitive functioning.

From the mediators tested in the present study, education had clearly the greatest mediating effect in the association of childhood SEP with the level of cognitive performance. It is well known that socioeconomic resources of the household are strong predictors of educational attainment of children^26^. The impact of childhood socioeconomic resources on education may differ by sex and regional context—in the less meritocratic European countries, children from less advantaged background may have been less likely to receive high educational attainment than children from more advantaged backgrounds and men may have gotten a greater access to education than women. Therefore, it is plausible that the mediating effect of education may differ by sex. This study suggests that education mitigates the later life cognitive risks related to early life conditions to almost the same degree for women and for men, with only a small difference, yet with statistical significance, which might be largely driven by the great sample size. Previous literature indicates that women gain a larger benefit from education than men in counteracting adverse childhood SEP^27^, which has been previously studied in the context of depression^28^. The theory of resource substitution proposed by Ross & Mirowsky proposes an explanation for this sex discrepancy^13^. The theory implies that access to multiple resources reduces the consequences of deficits of any particular resource. In comparison to men, women generally have less power, authority, independence, and earnings, so education can substitute the lack of these resources^13^. Therefore, education may provide stronger protection toward the cognitive health of women with lower childhood SEP as it substitutes for a deficit of alternative resources, which men are less dependent on. Given the greater burden of cognitive disorders in women, our results suggest that education can have in total a greater effect on cognitive health of women in the population, however, its strength as a mediator is limited for both women, and narrowly more for men.

Findings related to sex differences in the effect of childhood SEP on the rate of cognitive decline are less clear as we found the association between childhood socioeconomic disadvantage and the rate of cognitive decline only in one of the three cognitive measures. Nevertheless, our study suggests that the consequences of childhood socioeconomic disadvantage extend beyond disproportionately lower levels of cognitive performance for women, affecting trajectories of cognition, again more strongly for women than men, but only for delayed recall. This contributes new findings to the mixed literature concerning the effects of childhood SEP on the rate of cognitive decline. The majority of studies from the USA and Europe report no association between childhood SEP and the rate of cognitive decline^4,29–34^. However, some authors suggest that higher childhood SEP is associated with lower rates of cognitive decline^35^, while others report that childhood socioeconomic adversities are associated with lower rates of decline in some cognitive functions^5,36^. Sex differences, however, were rarely assessed^24,37^. Lyu and colleagues assessed cognition measured by word recall tests in individuals aged 65 or over from the Health and Retirement Study and found that being well-off during childhood was associated with steeper cognitive decline in men but not women^24^. Zaninotto and colleagues studied individuals aged 50 + years from the English Longitudinal Study of Ageing and found poor childhood SEP was associated with lower rates of decline in memory as well as cognitive functions only in women^37^. Considering the robustness of our sample, we conclude that exposure to childhood socioeconomic disadvantage, particularly in women, may weaken one’s ability to use pre-existing cognitive processes or compensatory mechanisms in coping with cumulating neuropathology in order to slow down cognitive decline.

While the effect sizes of the association of childhood SEP with cognition are small and the statistically significant results may be driven by the great sample size, the magnitude of the impact of low SEP on later life cognitive outcomes is notable on the population level. These findings are meaningful in the context of Europe which is facing challenges related to demographic shifts including increased lifespans^38^, and an increasing burden of chronic noncommunicable diseases, including dementia^39^. The total costs of informal care for dementia in Europe was 33 billion € in 2008, and the demographic forecast of costs is estimated to increase in Europe by about 43% between 2008 and 2030 to over 250 billion €^40^. Prevention policies for cognitive decline are vastly neglected, considering health systems across the OECD spend less than 3% of funds on prevention strategies^41^. A shift towards balancing primary, secondary and tertiary prevention of cognitive decline has the potential to not only reduce the risk for cognitive decline of an ageing population but could produce monumental decreases in the rate, burden and cost of care for older adults across Europe. Interventions preventing and targeting individuals with low childhood SEP and improving education could greatly mitigate the risks of social inequalities and improve the prognosis of later life cognitive health, particularly for women, who are the most affected by it.

Several limitations need to be mentioned. Data related to the childhood SEP were collected retrospectively, which is susceptible to recall bias. However, good internal and external consistency of SHARELIFE data has been demonstrated^42^. Moreover, results from the sensitivity analysis, in which we used alternative measure of SEP, support our main results. Additionally, there is a potential for selection bias. Growing up in socioeconomic hardship is linked to a higher cardiovascular mortality in later life^43^, therefore healthier people might be overrepresented in the study. This could lead to an underestimation of the associations that we found. Another limitation of the study is residual confounding and mediation. Other potential genetic, clinical and social risk factors, such as presence of APOE-ε4 allele, high blood pressure, high cholesterol levels or social isolation, were not included in the dataset and therefore are unaccounted for. In addition, we lack a high-quality measure of innate cognitive abilities, which is another important source of unmeasured confounding. Finally, it has been reported that women outperform men in verbal tasks, while men score higher in visuospatial tests^44^. Given that all three cognitive measures included in our study tested verbal abilities, our findings might be restricted to this cognitive domain only. Despite these limitations, to the best of our knowledge, this study is the largest longitudinal, population-based study with uniform data from the majority of European countries assessing sex differences in the role of childhood SEP in cognitive ageing.

This study adds to the evidence surrounding the link between childhood socioeconomic conditions and adult development and health, a complicated research challenge^38^. These findings are important in the context of identifying strategies to improve cognitive functioning of older populations, in particular addressing the disproportionately higher burden of cognitive impairment in women^2^. Unearthing the negative implications of low SEP on cognitive performance as more harmful to women than men provides a foundation for further research. Future studies should investigate the surrounding mechanisms driving these differences specifically focusing on educational and socioeconomical interventions for women with low childhood SEP. In poverty reduction initiatives, targeting especially young people and women could reduce the risk of later life problems. In education, providing additional support to low-income families and ensuring girls with low SEP are prioritized could improve individual prospects for ageing outcomes. Through targeting childhood SEP, prevention of cognitive decline in later life could be shifted to an early age potentially also increasing the impact on other risk factors across the life span. The emphasis on improving access to and achievement in education, particularly for girls and women, may be a worthwhile investment in reducing the burden of disability related to cognitive disorders of the ageing populations of Europe.

## Methods

### Source of data

Analysis is based on data from Survey of Health, Ageing and Retirement in Europe (SHARE), a multidisciplinary, cross-national study that collects information about health, social network and economic conditions of the ageing populations in 20 European countries and Israel. Data are collected using computer-assisted personal interviewing (CAPI), as previously described in detail^45^. The first wave of data collection was conducted in 2004 and was followed by five subsequent waves in 2006/2007 (wave 2), 2008/2009 (wave 3), 2011/2012 (wave 4), 2013 (wave 5), 2015 (wave 6) and 2017 (wave 7). Eligible participants were the oldest members of the household (aged at least 50 + years old) and their partners (if they had any), irrespective of age.

Only community-dwelling individuals were selected at baseline, but follow-up interviews were conducted in institutions, if a participant was admitted to one. New individuals were enrolled with the same sampling method in order to maintain the original age structure and because of attrition due to death and loss between waves. SHARE has been repeatedly reviewed and approved by the Ethics Committee of the University of Mannheim and the research was performed in accordance with relevant guidelines and regulations. All participants signed written informed consent and have been informed about the storage and use of data and their right to withdraw informed consent. All data were pseudo-anonymized. In addition, our study was approved by the Ethics Committee of the National Institute of Mental Health, Czech Republic.

### Childhood SEP

Wave 3 of SHARE was devoted to collecting information about participants’ life histories (SHARELIFE). In order to improve recollection process, a Life History Calendar was used for data collection^46^. To mitigate the risk of recall bias, a validation study of retrospectively recalled data in SHARELIFE has been previously carried out^42^. A shorter version of SHARELIFE was delivered in waves 5 and 7 to participants who had not completed this questionnaire in wave 3. Childhood SEP was operationalized through a composite variable of two household characteristics representing key components of wealth^47,48^. The composite variable “childhood SEP” includes two common indicators of household characteristics, which have been frequently used as they capture material circumstances related to SEP^48,49^: overcrowding, which in particular indicates socioeconomic hardship, and the number of books at home, capturing the intellectual aspects of SEP^47,48,50^.

First, participants were asked to recall two indicators of their household characteristics at the age of 10 years: (1) how many rooms their household occupied in their accommodation; (2) and how many members occupied their household. Second, they were asked how many books were in the place they lived: none or very few (0–10 books)/enough to fill one shelf (11–25 books)/enough to fill one bookcase (26–100 books)/enough to fill two bookcases (101–200 books)/enough to fill two or more bookcases (more than 200 books). The composite variable “childhood SEP” was created in two steps. First, we divided the number of rooms by the number of household members and created a “household ratio”, where higher values indicate a less crowded household. Then, we coded the variable “number of books” into a scale (from 0 to 4). Next, both variables “household ratio” and “number of books” were converted into z-scores. The average from these is the composite childhood SEP, where a lower value indicates a more disadvantaged SEP.

To assess the effects of childhood SEP on the rate of cognitive decline, we additionally created two binary variables specific for each country, corresponding to the most extreme distributions of childhood SEP—“childhood socioeconomic disadvantage” and “childhood socioeconomic advantage”. Childhood socioeconomic disadvantage was operationalized by fulfilling two conditions: (1) not reaching the household ratio above the 5th percentile in each country and (2) being in the most adverse category of number of books. Similarly, childhood socioeconomic advantage was defined by being above the 95th percentile of the household ratio in each country as well as being in the best or the second category of number of books, depending on the distribution in each country.

### Cognition

Assessment of cognition was conducted in six waves (wave 1, 2, 4, 5, 6 and 7) using three measures: verbal fluency, immediate recall and delayed recall. For cross-sectional analysis, data on cognition comes from the wave when all three cognitive measures were available for the first time, i.e. the wave differs for each participant. For longitudinal analysis, all available data on cognition was used. Verbal fluency score was derived from the animal fluency test^51^ and indicates the sum of acceptable animals that the participants could name within one minute. Immediate and delayed recall were extracted from the adapted 10-word delay recall test^52^. Immediate recall score (ranging from 0 to10) was measured as the number of recalled words after the interviewer read a list of 10 words. At the end of the cognitive testing session, interviewers asked participants to recall any of the words from the previous list, representing delayed recall score (ranging from 0 to10).

The association of SEP with the level of cognitive performance was similar for all three cognitive measures. Therefore, for the cross-sectional analysis, we transformed the three cognitive measures into z-scores and used their average as the composite measure of the level of cognitive performance. However, the association of childhood socioeconomic advantage/disadvantage with the rate of cognitive decline differed for each cognitive measure, therefore, we analysed them separately in the longitudinal analysis. Familiarity with the process of measuring cognitive functions and the test-specific content might be a cause of practice effect, leading to an underestimation of the rate of cognitive decline^53^. Thus, we adjusted the longitudinal analysis for practice effect. To select the best indicator of practice effect, we compared three sets of models adjusting for practice effect defined as (1) wave 1 set as indicator (2) number of prior tests and (3) root square of the number of prior tests^54^. The number of prior tests was the best fit and is therefore used in all models.

### Covariates

Covariates were identified based on previous evidence on the association of sociodemographic and health-related factors with childhood SEP and cognition^4,55^. Covariate data were derived from the same wave when cognition was measured or, if not available, then from the closest wave. Sociodemographic characteristics were age (years), sex (women vs. men), years of education, household net worth (standardized difference between household gross financial assets and financial liabilities), current working status (working vs. not working), cohabitation status (living with a partner vs. alone) and number of children and grandchildren.

Health-related characteristics were depressive symptoms (measured by EURO-D scale^56^), number of limitations in instrumental activities of daily living, cardiovascular disease (defined as ever diagnosed or treated for coronary heart disease, stroke, diabetes, high blood pressure or high blood cholesterol), number of chronic diseases, body mass index, physical inactivity (never vigorous nor moderate physical activity vs. physical activity), smoking (ever smoked daily vs. never smoked daily), alcohol use (drinking more than 2 glasses of alcohol almost every day vs. drinking less) and maximal grip strength. In addition, principal components analysis was used to explore whether five measures of physical health (i.e., number of chronic diseases, cardiovascular disease, number of limitations in instrumental activities of daily living, physical inactivity, and maximal grip strength) could measure an overall composite variable “physical state”. The established factor was utilized as a mediating latent factor in the structural equation model, for details see Supplementary Table S5.

### Analytical sample

From the 110,516 individuals who participated in the 3rd, 5th or 7th wave, we excluded those with missing data on childhood SEP (n = 3880) and cognitive functions (n = 19,523) as well as individuals younger than 50 years (n = 3054), leaving 84,059 persons in the final analytical sample used for the cross-sectional analysis (55% women; on average 64 years old). The study participants were from 4 European regions (Western Europe n = 33,294; Central and Eastern Europe n = 21,848; Southern Europe n = 16,966 and Scandinavia n = 9594) and Israel (n = 2357). Individuals with missing data on covariates (n = 2897) were kept in the sample for the descriptive analyses. For the longitudinal analysis, we included individuals with available measures on cognition in at least 2 waves (n = 74,279; 55% women). At baseline, they were on average 64 years old and were followed-up for the median of 2 130 days (approximately almost 6 years). Selection of the analytical sample is described in detail on Supplementary Fig. S4.

### Statistical analysis

The analysis was performed in several steps. First, we estimated whether the association of childhood SEP with the level of cognitive performance differs by sex (*cross-sectional analysis*). Second, we tested whether education, depressive symptoms and “physical state” mediate the association of childhood SEP with the level of cognitive performance differently for men and women (*mediation analysis*). Third, we studied whether men and women differ in the association of childhood socioeconomic advantage or disadvantage with the rate of cognitive decline (*longitudinal analysis*).

#### Cross-sectional analysis

Descriptive data of the analytical sample is presented as frequency (n [%]), mean ± standard deviation, or median and interquartile range. The sample size is large enough to assume normality^57^, therefore, differences in measurements between men and women were tested using independent samples t-test for continuous variables and χ2 test for binary variables. Because individuals (level 1) in our data were nested in countries (level 2), multilevel linear regression was applied to estimate beta coefficients with 95% confidence intervals (CIs) for the associations of childhood SEP with the level of cognitive performance. To examine whether there are cross-country differences in cognitive performance, an intercept-only model with a random intercept for country was compared with the null-model, using a likelihood ratio test. The formula for a regression equation for a random intercept-only model is as follows:

$$ {\text{Y}}_{{{\text{ij}}}} = \, \beta_{0} + {\text{ u}}_{{0{\text{j}}}} + {\text{ e}}_{{{\text{ij}}}} $$

In this formula u_0j_ is the between-country variance and e_ij_ is the within-country variance. The intra-class correlation coefficient was used to estimate the percentage of the total variance that can be attributed to differences between countries. Next, a random slope for SEP on country level was added to examine whether there are cross-country differences in the association of SEP with the level of cognitive performance, using a likelihood ratio test. The regression equation for a random intercept and a random slope model can be written as followed:

$$ {\text{Y}}_{{{\text{ij}}}} = \, \beta_{0} + \, \beta_{{1}} {\text{X}}_{{{\text{ij}}}} + {\text{ u}}_{{0{\text{j}}}} + {\text{ u}}_{{{\text{1j}}}} {\text{X}}_{{{\text{ij}}}} + {\text{ e}}_{{{\text{ij}}}} $$

Four sets of models are presented, step-wise adjusting for covariates. As the association of childhood SEP with the level of cognitive performance was consistent across all three cognitive measures (verbal fluency, immediate recall and delayed recall; data not presented in tables), results are presented only for the composite cognitive score.

Model 1 was adjusted for age and sex; Model 2 also for education; Model 3 also for depressive symptoms and sociodemographic characteristics (household net worth, cohabitation status, number of children, number of grandchildren and current working status); and Model 4 additionally for health-related characteristics (body mass index, number of limitations in instrumental activities of daily living, maximal grip strength, physical inactivity, number of chronic diseases, smoking status, cardiovascular disease and alcohol use).

Variance inflation factor (VIF) was calculated using simple linear regression model with all factors included in Model 4 to investigate the presence of multicollinearity (mean VIF = 1.53). Finally, to explore whether the association of childhood SEP with the level of cognitive performance differs by sex, an interaction term was included between sex and childhood SEP in all models. Likelihood ratio test assessed the interaction effect. As the interaction was significant in all models (*p* from < 0.001), all analyses were performed stratified by sex.

#### Mediation analysis

The strongest predictors of the level of cognitive performance were entered into a structural equation model with multiple mediations. Childhood SEP predicted the level of cognitive performance, mediated by years of education, depressive symptoms, and the latent “physical state” factor. “Physical state” was included into the model as an underlying latent factor predicting the five observed measures of physical health. Age predicted depressive symptoms, “physical state”, and cognitive performance and correlated with childhood SEP and years of education (Fig. 1). Men and women were tested separately, and their corresponding coefficients were compared using t-test. Because of cardiovascular disease and physical inactivity being binary outcome variables, Weighted Least Square Mean and Variance estimator was used. Model fit against the data was accepted if the root mean square error of approximation was lower than 0.08; and if the comparative fit index and Tucker–Lewis index were higher than 0.90^58^.

#### Longitudinal analysis

Linear mixed-effects models with unstructured covariance were used to study the relation of childhood SEP to the rate of cognitive decline. The basic three-level model was constructed as follows:

$$ {\text{Y}}_{{{\text{ijk}}}} = \, \beta_{0} + \, \beta_{{1}} {\text{X}}_{{{\text{ij}}.}} \left( {{\text{time}}_{{{\text{ij}}}} } \right) \, + {\text{ u}}_{{{\text{i}}..}} + {\text{ u}}_{{0{\text{ij}}.}} \, + {\text{ u}}_{{{\text{1j}}}} {\text{X}}_{{{\text{ij}}.}} \left( {{\text{time}}_{{{\text{ij}}}} } \right) \, + {\text{ e}}_{{{\text{ijk}}}} $$

As the association of childhood SEP with the rate of cognitive decline is different for various cognitive measures^5^, the three cognitive measures (verbal fluency, immediate recall and delayed recall) were analysed separately. They were entered into the models in their original form, not as standardized scores. The models included time (in years since baseline centred around grand mean), time squared (in years), childhood SEP and their interaction term (childhood SEP × time), adjusting for baseline age, sex and practice effect. To explore whether the association of childhood SEP on cognitive decline differs by sex, an interaction term was included between time, childhood SEP and sex (childhood SEP × time × sex) in the model. As there was a significant interaction with sex for all the three cognitive measures (*p* from likelihood ratio test < 0.001 for all three measures), all analyses were performed stratified by sex.

To enable interpretation of the effect of childhood SEP, binary variables (childhood socioeconomic disadvantage and childhood socioeconomic advantage) were used. The models then included time, childhood socioeconomic advantage/disadvantage and their interaction term (childhood socioeconomic advantage/disadvantage × time), adjusting for baseline age. Participants, time in years and countries were set as random intercepts, the time squared as random slope at participant level and also as fixed effect, childhood socioeconomic advantage/disadvantage as random slope at country level and also fixed effect, and baseline age as fixed effect in Model 1. In a step-wise approach, other covariates were added as fixed effects: education in Model 2; depressive symptoms and sociodemographic characteristics (household net worth, cohabitation status, number of children, number of grandchildren and current working status) in Model 3; health-related characteristics (body mass index, limitations in instrumental activities of daily living, maximal grip strength, physical inactivity, number of chronic diseases, smoking status, cardiovascular disease, alcohol use) in Model 4.

#### Sensitivity analysis

To test the robustness of our findings, we performed a sensitivity analysis using an alternative independent variable characterizing childhood SEP, as used in previous research^59^. The alternative childhood SEP was the sum score of four binary indicators of socioeconomic conditions at the age of 10: (1) the main breadwinner’s occupational position (low skilled), (2) number of books at home (less than 10), (3) overcrowding (more than 1 person per 1 room), and (4) poor housing quality (lacking all of the following features: fixed bath, cold running water supply, hot running water supply, inside toilet, and central heating). As all these measures were collected only in wave 3, the sensitivity analysis was performed only on a subsample of 25 574 individuals. Methods of multiple linear regression were used to build 4 models adjusting for covariates.

In the end, the multiple mediation model was run using the “physical inactivity” and the “alcohol use” variables instead of the “physical state” latent factor to see if more specific behavioural recommendations could be made based on the mediation model. Analyses were conducted using STATA software, version 16.1 and Mplus Ver. 8.

## Supplementary Information

Supplementary Information.

## Data Availability

Access to the SHARE data is free of charge based on individual registration. Details can be found on www.share-project.org. The study protocol and syntax of the statistical analysis can be shared upon request from the corresponding author of this study.

## References

[1] Winblad B (2016). Defeating Alzheimer's disease and other dementias: a priority for European science and society. Lancet Neurol..

[2] Gao S, Hendrie HC, Hall KS, Hui S (1998). The relationships between age, sex, and the incidence of dementia and Alzheimer disease: a meta-analysis. Arch. Gen. Psychiatry.

[3] Moceri VM, Kukull WA, Emanuel I, van Belle G, Larson EB (2000). Early-life risk factors and the development of Alzheimer's disease. Neurology.

[4] Cermakova P, Formanek T, Kagstrom A, Winkler PJN (2018). Socioeconomic position in childhood and cognitive aging in Europe. Neurology.

[5] Aartsen MJ (2019). Advantaged socioeconomic conditions in childhood are associated with higher cognitive functioning but stronger cognitive decline in older age. Proc. Natl. Acad. Sci..

[6] Janicki-Deverts D, Cohen S, Matthews KA, Jacobs DR (2012). Sex differences in the association of childhood socioeconomic status with adult blood pressure change: the CARDIA study. Psychosom. Med..

[7] Wagner KJP, Bastos JLD, Navarro A, Gonzalez-Chica DA, Boing AF (2018). Socioeconomic status in childhood and obesity in adults: a population-based study. Rev. Saude Publ..

[8] Hyde JS (2014). Gender similarities and differences. Annu. Rev. Psychol..

[9] Voyer D (2011). Time limits and gender differences on paper-and-pencil tests of mental rotation: a meta-analysis. Psychon. Bull. Rev..

[10] Maeda Y, Yoon SY (2013). A meta-analysis on gender differences in mental rotation ability measured by the purdue spatial visualization tests: visualization of rotations (PSVT:R). Educ. Psychol. Rev..

[11] Wood W, Eagly AH (2012). Advances in Experimental Social Psychology.

[12] Else-Quest NM, Hyde JS, Linn MC (2010). Cross-national patterns of gender differences in mathematics: a meta-analysis. Psychol Bull.

[13] Ross CE, Mirowsky J (2006). Sex differences in the effect of education on depression: resource multiplication or resource substitution?. Soc. Sci. Med..

[14] Winchester SB, Sullivan MC, Roberts MB, Granger DA (2016). Prematurity, birth weight, and socioeconomic status are linked to atypical diurnal hypothalamic-pituitary-adrenal axis activity in young adults. Res. Nurs. Health.

[15] Ivy AS (2010). Hippocampal dysfunction and cognitive impairments provoked by chronic early-life stress involve excessive activation of CRH receptors. J. Neurosci. Off. J. Soc. Neurosci..

[16] Curran MM, Sandman CA, Davis EP, Glynn LM, Baram TZ (2017). Abnormal dendritic maturation of developing cortical neurons exposed to corticotropin releasing hormone (CRH): insights into effects of prenatal adversity?. Plus ONE.

[17] Sandman CA (2018). Cortical thinning and neuropsychiatric outcomes in children exposed to prenatal adversity: a role for placental CRH?. Am. J. Psychiatry.

[18] Lee, C., Ryff, C. D., & Coe, C. L. Gender, early life adversity, and adult health. *The Oxford Handbook of Integrative Health Science* (eds Ryff, C. D. & Krueger, R. F.) (Oxford University Press, 2018)

[19] Suglia SF (2018). Childhood and adolescent adversity and cardiometabolic outcomes: a scientific statement from the American heart association. Circulation.

[20] Hamil-Luker J, O’Rand AJD (2007). Gender differences in the impact of childhood adversity on the risk for heart attack across the life course. Demography.

[21] González D, Nazmi A, Victora CG (2009). Childhood poverty and abdominal obesity in adulthood: a systematic review. Cad. Saude Publ..

[22] Pudrovska T, Reither EN, Logan ES, Sherman-Wilkins KJ (2014). Gender and reinforcing associations between socioeconomic disadvantage and body mass over the life course. J. Health Soc. Behav..

[23] Hurst L (2013). Lifetime socioeconomic inequalities in physical and cognitive aging. Am. J. Public Health.

[24] Lyu J (2015). Gender Differences in the association between childhood socioeconomic status and cognitive function in later life. J. Geriatr..

[25] van der Flier WM, Scheltens P (2005). Epidemiology and risk factors of dementia. J. Neurol. Neurosurg. Psychiatry..

[26] Lawlor DA (2005). Childhood socioeconomic position, educational attainment, and adult cardiovascular risk factors: the Aberdeen children of the 1950s cohort study. Am. J. Public Health.

[27] Schaan B (2014). The interaction of family background and personal education on depressive symptoms in later life. Soc. Sci. Med..

[28] Csajbók Z (2021). Sex differences in the association of childhood socioeconomic position and later-life depressive symptoms in Europe: the mediating effect of education. Soc. Psychiatry Psychiatr. Epidemiol..

[29] Everson-Rose SA, Mendes de Leon CF, Bienias JL, Wilson RS, Evans DA (2003). Early life conditions and cognitive functioning in later life. Am. J. Epidemiol..

[30] Wilson RS (2005). Socioeconomic characteristics of the community in childhood and cognition in old age. Exp. Aging Res..

[31] Brown MT (2010). Early-life characteristics, psychiatric history, and cognition trajectories in later life. Gerontologist.

[32] González HM, Tarraf W, Bowen ME, Johnson-Jennings MD, Fisher GG (2013). What do parents have to do with my cognitive reserve? Life course perspectives on twelve-year cognitive decline. Neuroepidemiology.

[33] Staff RT, Chapko D, Hogan MJ, Whalley LJ (2016). Life course socioeconomic status and the decline in information processing speed in late life. Soc. Sci. Med..

[34] Ericsson M (2017). Childhood social class and cognitive aging in the Swedish adoption/twin study of aging. J. Proc. Natl. Acad. Sci..

[35] Marden JR, Tchetgen Tchetgen EJ, Kawachi I, Glymour MM (2017). Contribution of socioeconomic status at 3 life-course periods to late-life memory function and decline: early and late predictors of dementia risk. Am. J. Epidemiol..

[36] Barnes LL (2012). Effects of early-life adversity on cognitive decline in older African Americans and whites. Neurology.

[37] Zaninotto P, Batty GD, Allerhand M, Deary IJ (2018). Cognitive function trajectories and their determinants in older people: 8 years of follow-up in the english longitudinal study of ageing. J. Epidemiol. Commun. Health.

[38] Suzman R, Beard J (2011). Global health and aging. NIH Publ..

[39] Sleeman KE (2019). The escalating global burden of serious health-related suffering: projections to 2060 by world regions, age groups, and health conditions. Lancet Glob. Health.

[40] Wimo, A. & Jonsson, L. J. Cost of Illness and Burden of Dementia in Europe Prognosis to 2030. *Alzheimer Eur*. (2009).

[41] OECD. *Addressing Dementia: The OECD Response*. (OECD Publishing, Paris, 2015).

[42] Havari E, Mazzonna F (2015). Can we trust older people’s statements on their childhood circumstances? Evidence from SHARELIFE. Eur. J. Popul..

[43] Stringhini S (2018). Socio-economic trajectories and cardiovascular disease mortality in older people: the english longitudinal study of ageing. Int. J. Epidemiol..

[44] Little BR (1969). Sex differences and comparability of three measures of cognitive complexity. Psychol. Rep..

[45] Börsch-Supan A (2013). Data resource profile: the survey of health, ageing and retirement in Europe (SHARE). Int. J. Epidemiol.

[46] Freedman D, Thornton A, Camburn D, Alwin D, Young-DeMarco LJ (1988). The life history calendar: a technique for collecting retrospective data. Soc. Method..

[47] Galobardes B, Shaw M, Lawlor DA, Lynch JW (2006). Indicators of socioeconomic position (part 2). J. Epidemiol. Commun. Health.

[48] Galobardes B (2006). Indicators of socioeconomic position (part 1). J. Epidemiol. Commun. Health.

[49] Niedzwiedz CL, Katikireddi SV, Pell JP, Mitchell R (2014). The association between life course socioeconomic position and life satisfaction in different welfare states: European comparative study of individuals in early old age. Age Ageing.

[50] Winkler P, Formánek T, Mladá K, Cermakova P (2018). The CZEch mental health study (CZEMS): study rationale, design, and methods. Int. J. Methods Psychiatric Res..

[51] Henley NM (1969). A psychological study of the semantics of animal terms. J. Verbal Learn. Verbal Behav..

[52] Harris SJ, Dowson JH (1982). Recall of a 10-word list in the assessment of dementia in the elderly. Br. J. Psychiatry.

[53] Weuve J (2015). Guidelines for reporting methodological challenges and evaluating potential bias in dementia research. Alzheimers Dement.

[54] Vivot A (2016). Jump, hop, or skip: modeling practice effects in studies of determinants of cognitive change in older adults. Am. J. Epidemiol..

[55] Formanek T, Kagstrom A, Winkler P, Cermakova P (2019). Differences in cognitive performance and cognitive decline across European regions: a population-based prospective cohort study. Eur. Psychiatry J. Assoc. Eur. Psychiatr..

[56] Organization for Economic and Development. *Classifying Educational Programmes: Manual for ISCED-97 Implementation in OECD Countries*. (OECD Paris, 1999).

[57] Prince MJ (1999). Development of the EURO–D scale–a European Union initiative to compare symptoms of depression in 14 European centres. Br. J. Psychiatry.

[58] Brown TA (2014). Confirmatory Factor Analysis for Applied Research.

[59] Wahrendorf M, Blane D (2015). Does labour market disadvantage help to explain why childhood circumstances are related to quality of life at older ages? Results from SHARE. Aging Mental Health.

