# Supplementary material for: Role of sex in the association between childhood socioeconomic position and cognitive ageing in later life
Source: Sci Rep. 2021 Feb 25;11:4647. doi: 10.1038/s41598-021-84022-1 (PMC7907064; doi:10.1038/s41598-021-84022-1)
Supplement: Supplementary file 1 — Supplementary Information. [file 41598_2021_84022_MOESM1_ESM.docx]

**Role of sex in the association between childhood socioeconomic position and cognitive ageing in later life**

Katrin Wolfova, MD^a,b^, Zsofia Csajbok, MA^a,b,c^, Anna Kagstrom, MSc^a,b^, Ingemar Kåreholt, PhD^d,e^, Pavla Cermakova, MD, PhD^a,b,f*^

*^a^ Department of Psychiatry and Medical Psychology, Third Faculty of Medicine, Charles University in Prague, Ruska 87, 100 00 Prague, Czech Republic*

*^b^ National Institute of Mental Health, Topolova 748, 250 67 Klecany, Czech Republic*

*^c^ Department of Philosophy and History of Science, Faculty of Science, Charles University in Prague, Vinicna 7, 128 00 Prague, Czech Republic*

*^d^  Institute of Gerontology, Aging Research Network - Jönköping (ARN-J), School of Health and Welfare, Jönköping University, Jönköping, Sweden*

*^e^ Aging Research Center, Karolinska Institutet and Stockholm University, Stockholm, Sweden*

*^f^ Department of Epidemiology, Second Faculty of Medicine, Charles University in Prague, Plzenska 130/221, 150 00 Prague, Czech Republic*

***ORCID of the authors:***

*Katrin Wolfova: 0000-0003-0295-8848*

*Zsofia Csajbok: 0000-0002-5800-1597*

*Anna Kagstrom: 0000-0002-1455-1300*

*Ingemar Kåreholt: 0000-0002-8617-0355*

*Pavla Cermakova: 0000-0002-9282-9363*

**Correspondence to:**

Pavla Cermakova, MD, PhD

Third Faculty of Medicine

Charles University

Ruská 87, 100 00 Prague 10

Czech Republic

[Pavla.Cermakova@nudz.cz](mailto:Pavla.Cermakova@nudz.cz)

+420 283 088 405

**SUPPLEMENTARY MATERIAL**

**Supplementary Table S1** Association of childhood socioeconomic position with the level of cognitive performance in the whole analytical sample (n=84 059)

|  | **B (95% CI)** | **p value** |
| --- | --- | --- |
| **Model 1** |  |  |
| SEP | 0.223 (0.194; 0.251) | <0.001 |
| Between-country variance | 0.056 |  |
| Within-country variance | 0.478 |  |
| **Model 2** |  |  |
| SEP | 0.132 (0.104; 0.161) | <0.001 |
| Between-country variance | 0.045 |  |
| Within-country variance | 0.447 |  |
| **Model 3** |  |  |
| SEP | 0.120 (0.092; 0.148) | <0.001 |
| Between-country variance | 0.042 |  |
| Within-country variance | 0.431 |  |
| **Model 4** |  |  |
| SEP | 0.114 (0.088; 0.141) | <0.001 |
| Between-country variance | 0.035 |  |
| Within-country variance | 0.412 |  |

*CI=confidence interval; SEP=socioeconomic position*

*Model 1: adjusted for age and sex*

*Model 2: adjusted for age, sex and education*

*Model 3: adjusted for age, sex, education, depressive symptoms and socioeconomic characteristics (household net worth, cohabitation status, number of children, number of grandchildren, current working status)*

*Model 4: adjusted for age, sex, education, socioeconomic characteristics (household net worth, cohabitation status, number of children, number of grandchildren, current working status) and health related characteristics (body mass index, depressive symptoms limitations with instrumental activities of daily living, maximum of grip strength, physical inactivity, number of chronic diseases, smoking status, cardiovascular disease and alcohol use)*

**Supplementary Table S2** Beta coefficients for the association of all variables with the level of cognitive performance

|  | **Women**  **(n=44 269)** | **Men**  **(n=36 893)** | | |
| --- | --- | --- | --- | --- |
|  | **B (95% CI)** | **β** | **B (95% CI)** | **β** |
| Childhood SEP | 0.159 (0.183; 0.204)*** | 0.152 | 0.137 (0.128; 0.147)*** | 0.140 |
| Age | -0.016 (-0.017; -0.015)*** | -0.183 | -0.014 (-0.015; -0.013)*** | -0.170 |
| Years of education | 0.047 (0.046; 0.049)*** | 0.240 | 0.040 (0.038; 0.042)*** | 0.224 |
| Highest decile of household net worth | 0.078 (0.056; 0.100)*** | 0.027 | 0.066 (0.044; 0.088)*** | 0.026 |
| Current working status | 0.094 (0 .077; 0.111)*** | 0.051 | 0.037 (0.019; 0.056)*** | 0.023 |
| Number of children | -0.002 (-0.008; 0.004) | -0.003 | 0.002 (-0.004; 0.009) | 0.004 |
| Number of grandchildren | 0.005 (0.002; 0.008)** | 0.017 | >0.001 (-0.003; 0.004) | 0.002 |
| Living with a partner | 0.016 (0.002; 0.030)* | 0.009 | 0.030 (0.012; 0.047)** | 0.015 |
| Limitations in IADL | -0.069 (-0.077; -0.061)*** | -0.077 | -0.076 ( -0.085; -0.067)*** | -0.079 |
| Depressive symptoms | -0.033 (-0.036; -0.030)*** | -0.092 | -0.042 ( -0.046; -0.038)*** | -0.104 |
| Chronic diseases | 0.033 (0.028; 0.038)*** | 0.062 | 0 .027 (0.021; 0 .033)*** | 0.051 |
| CVD | -0.061 (-0.076; -0.045)*** | -0.036 | -0.037 (-0.054; -0.020)*** | -0.023 |
| Body mass index | -0.002 (-0.003; -0.001)** | -0.011 | -0.003 (-0.005; -0.002)*** | -0.017 |
| Physical inactivity | -0.156 (-0.180; -0.133)*** | -0.054 | -0.112 (-0.140; -0.084)*** | -0.036 |
| Smoking | 0.076 (0.062; 0.089)*** | 0.042 | -0.019 (-0.033; -0.005)** | -0.012 |
| Alcohol use | 0.028 (0.006; 0.050)* | 0.010 | 0.001 (-0.015; 0.016) | >0.001 |
| Maximal grip strength | 0.016 (0.015; 0.017)*** | 0.139 | 0.013 (0.012; 0.013)*** | 0.164 |

* p < 0.05, ** p < 0.01, *** p < 0.001.

*CI=confidence interval; IADL=instrumental activities of daily living; CVD=cardiovascular disease; B=unstandardized beta; β=standardized beta. All variables in the table were entered into the model simultaneously.*

**Supplementary Table S3** Results of the multiple mediation model expressed in the effect on the level of cognitive performance and percentage in relation to total effect across sex

|  | **Percentage of the mediating effect in relation to the total effect** | | **Effect on cognition (SE)** | | **Effect size of sex difference** |
| --- | --- | --- | --- | --- | --- |
| **Accounted paths** | **Men** | **Women** | **Men** | **Women** | **Cohen’s d** |
| Total Effect ^A^ (childhood SEP to cognition) | 100% | 100% | 0.261 (0.003) | 0.316 (0.007) | 0.052*** |
| Education ^A^ | 29.50% | 30.70% | 0.077 (0.002) | 0.097 (0.002) | 0.051*** |
| Depressive Symptoms ^A^ | N.A. | N.A. | -0.002 (0.000) | -0.002 (0.001) | 0.000 |
| Physical State ^A^ | 14.56% | 14.87% | 0.038 (0.003) | 0.047 (0.002) | 0.018** |
| Education 🡪 Depressive Symptoms ^A^ | N.A. | N.A. | -0.001 (0.000) | -0.001 (0.000) | 0.000 |
| Education 🡪 Physical State ^A^ | 8.43% | 8.54% | 0.022 (0.001) | 0.027 (0.001) | 0.026*** |
| Childhood SEP direct effect ^A^ | 48.28% | 46.84% | 0.126 (0.003) | 0.148 (0.002) | 0.044*** |
| Total Effect ^B^ (age to cognition) | 100% | 100% | -0.024 (0.000) | -0.027 (0.000) | 0.042*** |
| Depressive Symptoms ^B^ | 0.00% | 0.00% | 0.000 (0.000) | 0.000 (0.000) | 0.000 |
| Physical state ^B^ | 87.50% | 77.78% | -0.021 (0.001) | -0.021 (0.001) | 0.000 |
| Age direct effect ^B^ | 12.50% | 22.22% | -0.003 (0.001) | -0.006 (0.000) | -0.023** |

* p < 0.05, ** p < 0.01, *** p < 0.001.

*^A^ Paths directly and mediating between childhood SEP and cognition.*

*^B^ Paths directly and mediating between age and cognition.*

*SEP = socioeconomic position; SE = standard error. N.A. = not applicable is used when the percentage of the mediating effect related to the total effect is not interpreted because the mediating effect is negative in contrast to the total effect being positive.*

**Supplementary Table S4** Association of childhood socioeconomic position with the level of cognitive performance in women and men using alternative definition of childhood socioeconomic position

|  | | | | | | | **B (95% CI)** | | | | | | | |
| --- | --- | --- | --- | --- | --- | --- | --- | --- | --- | --- | --- | --- | --- | --- |
|  | | | **Women**  (n=13 996) | | | | **Men**  (n=11 578) | | | **Interaction childhood SEP × sex in the whole sample** | | | | |
| Childhood SEP (ref. most advantaged) | | |  | | | |  | | |  | | | | |
| **Model 1** |  | | | |  | | | | | | |  | | |
| Advantaged | | | -0.181 (-0.235; -0.126)*** | | | | -0.137 (-0.193; -0.080)*** | | | -0.041 (-0.120; 0.038) | | | | |
| Middle | | | -0.345 (-0.397; -0.293)*** | | | | -0.276 (-0.330; -0.222)*** | | | -0.069 (-0.144; 0.006) | | | | |
| Disadvantaged | | | -0.652 (-0.705; -0.599)*** | | | | -0.537 (-0.592; -0.482)*** | | | -0.126 (-0.203; -0.050)** | | | | |
| Most disadvantaged | | | -0.899 (-0.954; -0.844)*** | | | | -0.708 (-0.765; -0.651)*** | | | -0.216 (-0.295; -0.138)*** | | | | |
| **Model 2** | | | |  | | |  | | | |  | | | |
| Advantaged | | | -0.076 (-0.127; -0.024)** | | | | -0.047 (-0.102; 0.007) | | | -0.045 (-0.120; 0.029) | | | | |
| Middle | | | -0.153 (-0.202; -0.103)*** | | | | -0.108 (-0.160; -0.056)*** | | | -0.081 (-0.152; -0.010)* | | | | |
| Disadvantaged | | | -0.317 (-0.369; -0.265)*** | | | | -0.260 (-0.314; -0.205)*** | | | -0.125 (-0.197; -0.053)** | | | | |
| Most disadvantaged | | | -0.446 (-0.502; -0.391)*** | | | | -0.334 (-0.392; -0.276)*** | | | -0.211 (-0.285; -0.136)*** | | | | |
| **Model 3** | | | |  | | |  |  | | | | |  | |
| Advantaged | | | -0.068 (-0.119; -0.017)** | | | | -0.050 (-0.103; 0.004) | | | -0.031 (-0.104; 0.042) | | | | |
| Middle | | | -0.143 (-0.192; -0.094)*** | | | | -0.104 (-0.156; -0.053)*** | | | -0.069 (-0.138; 0.001) | | | | |
| Disadvantaged | | | -0.292 (-0.343; -0.241)*** | | | | -0.243 (-0.297; -0.189)*** | | | -0.107 (-0.178; -0.036)** | | | | |
| Most disadvantaged | | | -0.415 (-0.471; -0.360)*** | | | | -0.319 (-0.376; -0.261)*** | | | -0.180 (-0.253; -0.107)*** | | | | |
| **Model 4** | | | |  |  | | |  | | | | | | |
| Advantaged | | | -0.059 (-0.109; -0.010)* | | | | -0.050 (-0.103; 0.003) | | | -0.018 (-0.091; 0.054) | | | | |
| Middle | | | -0.127 (-0.175; -0.079)*** | | | | -0.105 (-0.156; -0.054)*** | | | -0.047 (-0.116; 0.021) | | | | |
| Disadvantaged | | | -0.274 (-0.325; -0.223)*** | | | | -0.229 (-0.282; -0.175)*** | | | -0.100 (-0.171; -0.030)** | | | | |
| Most disadvantaged | | | -0.383 (-0.438; -0.328)*** | | | | -0.297 (-0.354; -0.240)*** | | | -0.174 (-0.247; -0.101)*** | | | | |

* p<0.05, ** p<0.01, *** p<0.001

*Results are unstandardized beta coefficients with 95% confidence intervals from linear regression models estimating the association of childhood socioeconomic position with cognition.*

*SEP=socioeconomic position; CI=confidence interval*

*Model 1: adjusted for age and country*

*Model 2: adjusted for age, country and education*

*Model 3: adjusted for age, country, education, depressive symptoms and socioeconomic characteristics (household net worth, cohabitation status, number of children, number of grandchildren and current working status)*

*Model 4: adjusted for age, country, sex, education, socioeconomic characteristics (household net worth, cohabitation status, number of children, number of grandchildren, current working status) and health related characteristics (body mass index, depressive symptoms, limitations with instrumental activities of daily living, maximum of grip strength, physical inactivity, number of chronic diseases, smoking status, cardiovascular disease and alcohol use)*

**Supplementary Table S5** Factor loadings of the variables in the “physical state” principal component

|  | Component |
| --- | --- |
|  | 1 |
| Zscore: Number of chronic diseases | 0.783 |
| Cardiovascular disease | 0.661 |
| Zscore: Limitations in instrumental activities of daily living | 0.614 |
| Physical inactivity | 0.511 |
| Zscore: maximal grip strength | -0.511 |
| A composite variable “physical state” was created using principal components analysis and utilized in a structural equation model. The variables entered into the model were body mass index, number of limitations in instrumental activities of daily living, maximal grip strength, cardiovascular disease, total number of chronic diseases, physical inactivity, smoking, and alcohol. All continuous variables were standardized. Body mass index, smoking, and alcohol did not have satisfactory loading (i.e., higher than 0.5) on neither the one-factor model, nor a multiple factor solution. After the omission of them, the remaining variables formed a satisfactory one-factor solution (i.e., all the variables had loaded higher than 0.5 on the sole Principal Component). The one principal component accounted for 39.02% of the data variance. | |


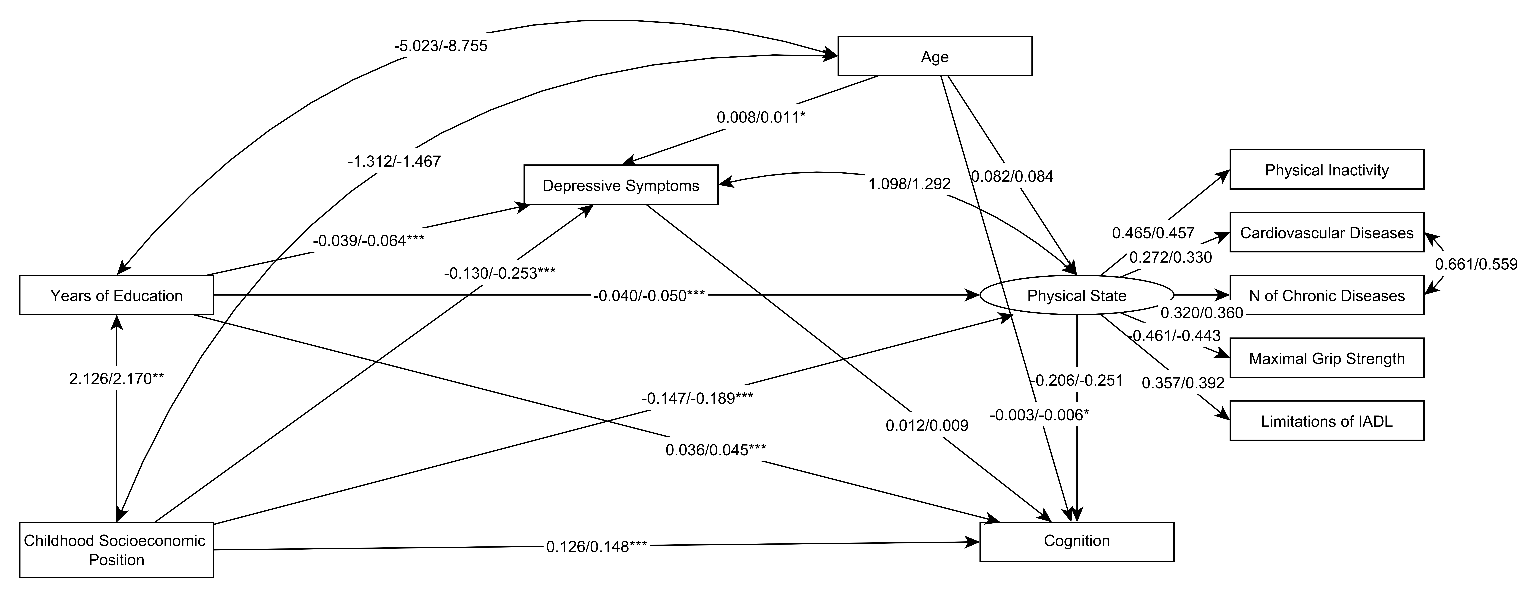


**Supplementary Figure S1** Multiple mediation model where one of the mediators is the latent Physical state factor

* p < 0.05, ** p < 0.01, *** p < 0.001.

The figure presents an analysis testing mediating effect of education, depressive symptoms, and physical state on the association between childhood socioeconomic position and cognition controlled for age across sex. Unstandardized coefficients are presented on their respective arrows. Men’s and women’s coefficients are on the left and right position of the slash, respectively. All the paths are significant at p < 0.001.

The paths significantly different between men and women are marked with respective asterisks.

**
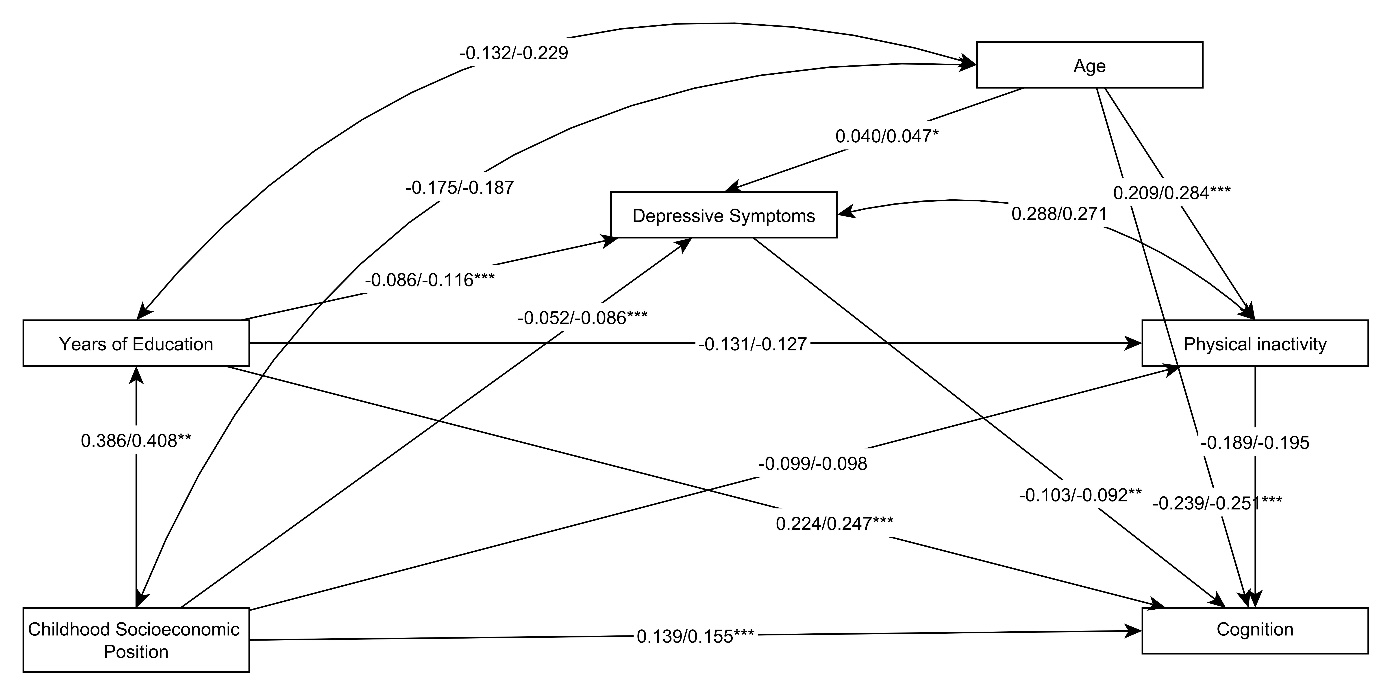
**

**Supplementary Figure S2** Multiple mediation model with Physical inactivity as a mediator

* p < 0.05, ** p < 0.01, *** p < 0.001.

The figure presents an analysis testing the mediating effect of education, depressive symptoms, and physical inactivity on the association between childhood socioeconomic position and cognition controlled for age across sex. Standardized coefficients are presented on their respective arrows. Men’s and women’s coefficients are on the left and right position of the slash, respectively. All the paths are significant at p < 0.001.

The paths significantly different between men and women are marked with respective asterisks.

The whole mediation model explained 30.2% and 37.2% of men’s and women’s variance in cognition, respectively. Physical inactivity alone meditated 7.2% and 6.3% of the total effect on cognition in men and women, respectively.

**
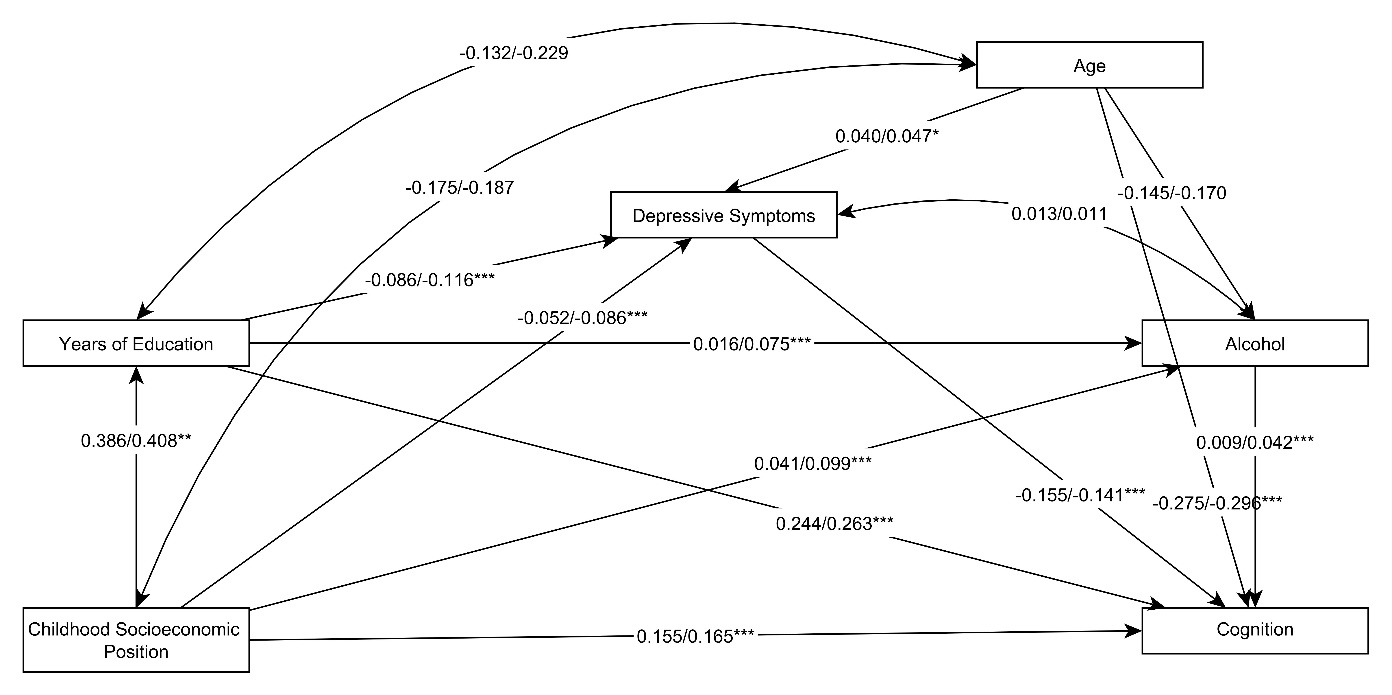
**

**Supplementary Figure S3** Multiple mediation model with alcohol use as a mediator

* p < 0.05, ** p < 0.01, *** p < 0.001.

The figure presents an analysis testing the mediating effect of education, depressive symptoms, and alcohol use on the association between childhood socioeconomic position and cognition controlled for age across sex. Standardized coefficients are presented on their respective arrows. Men’s and women’s coefficients are on the left and right position of the slash, respectively. All the paths are significant at p < 0.001, except the effect of alcohol on cognition in men which is nonsignificant, and the effect of education on alcohol in men which is only significant at p < 0.05.

The paths significantly different between men and women are marked with respective asterisks.

The whole mediation model explained 27.2% and 34.3% of men’s and women’s variance in cognition, respectively. Alcohol alone meditated 0% and 1.3% of the total effect on cognition in men and women, respectively.

**
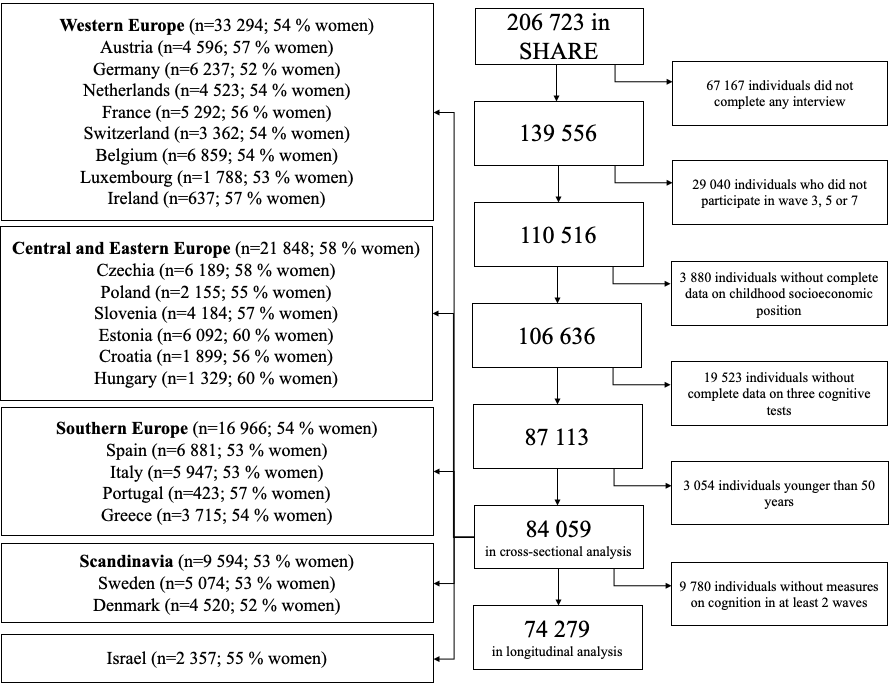
**

**Supplementary Figure S4** Selection of the analytical samples
